# First Record of Soft Tissue Preservation in the Upper Devonian of Poland

**DOI:** 10.1371/journal.pone.0142619

**Published:** 2015-11-11

**Authors:** Michał Zatoń, Krzysztof Broda

**Affiliations:** University of Silesia, Faculty of Earth Sciences, Będzińska 60, Sosnowiec, Poland; Université de Poitiers, FRANCE

## Abstract

Soft tissue preservation is reported from Upper Devonian deposits of the Holy Cross Mountains, central Poland, for the first time. The preserved soft tissues are muscles associated with arthropod cuticle fragments. The muscles are phosphatized with variable states of preservation. Well-preserved specimens display the typical banding of striated muscles. Other muscle fragments are highly degraded and/or recrystallized such that their microstructure is barely visible. The phosphatized muscles and associated cuticle are fragmented, occur in patches and some are scattered on the bedding plane. Due to the state of preservation and the lack of diagnostic features, the cuticle identification is problematic; however, it may have belonged to a phyllocarid crustacean. Taphonomic features of the remains indicate that they do not represent fossilized fecal matter (coprolite) but may represent a regurgitate, but the hypothesis is difficult to test. Most probably they represent the leftover remains after arthropod or fish scavenging. The present study shows that soft tissues, which even earlier were manipulated by scavenger, may be preserved if only special microenvironmental conditions within and around the animal remains are established.

## Introduction

Well-preserved fossils are widespread in both space and time, but those having soft tissues preserved are mainly limited to deposits that formed under specific environmental and geochemical conditions. Such deposits, termed Konservat-Lagerstätten [1], have a patchy global distribution [2–3]. With respect to marine paleoenvironments, the most frequent Lagerstätte deposits are confined to the oldest, Ediacaran (Neoproterozoic) and Cambrian periods, followed by those from the Ordovician and Devonian, and then from the Jurassic [3]. The fidelity of soft tissue preservation may also vary between different Lagerstätte deposits, between different fossils in the same deposits, and even between different parts of the same fossil [4].

Although the soft-bodied organisms and the animals' soft-tissues may be preserved through a variety of early diagenetic processes, such as replication by clay minerals [5–6], calcitization [7] or pyritization [8–10], the mineral most commonly preserving soft tissues is apatite [11]. Many soft-bodied organisms and structures are known to be exquisitely preserved due to phosphatization processes. These include such geologically old fossils as algae and animal embryos from the famous Ediacaran Doushantuo phosphorite of China [12–13], or tiny Cambrian arthropods from the 'Orsten' Lagerstätte of Sweden [14]. However, phosphatization processes frequently affected such labile soft parts as muscle tissues. Phosphatized muscles are known from many different invertebrate and vertebrate taxa throughout the Phanerozoic, including Silurian and Jurassic horseshoe crabs [15–16], Devonian and Jurassic fishes [17–19], Devonian and Jurassic crustaceans [4, 20–22], Jurassic marine reptiles [23], Cretaceous pterosaurs [24] and dinosaurs [25]. The iconic examples, however, are from the Cretaceous Santana Formation in Brazil where phosphatic soft tissues of fishes are excellently preserved at the subcellular level, retaining such structures as cell nuclei and mitochondria [26–28]. Due to phosphatization, the exceptionally preserved soft tissues not only provide a unique window into paleobiology of extinct organisms, but also gave the impetus for laboratory experiments which were designed to help understanding fossilization processes in the context of microenvironmental and geochemical conditions [29–33].

Here we report on the preservation of soft tissues in the form of phosphatized muscles from the Upper Devonian of the Holy Cross Mountains, central Poland, for the first time. Interestingly, the phosphatized soft tissues have been found in lower Famennian deposits containing abundant phosphatic arthropod cuticle and non-biomineralized algae. The deposits, generally coined as the Kowala Lagersttätte [34], however, have not provided any fossilized animal soft tissues so far. Therefore, this find indicates that the deposits are prospective for soft tissue preservation, suggesting this lower Famennian site may be another conservation Lagersttätte. Importantly, it is confined to the time-interval shortly post-dating the Frasnian-Famennian biotic crisis.

## Material and Methods

**Fig 1:**
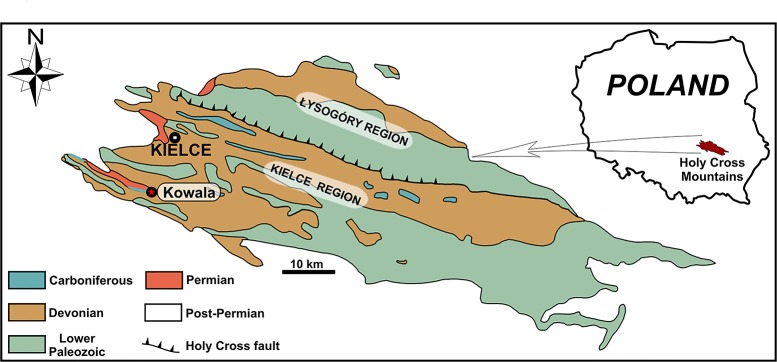
Geological sketch-map of the Paleozoic of the Holy Cross Mountains with the Kowala quarry indicated by the red star (modified after Marynowski et al. [51]).

The preserved soft tissues were found on the surface of a laminated, marly shale sample derived from the lower Famennian portion of a marine, Upper Devonian sequence exposed at the Kowala quarry in the Holy Cross Mountains, Poland (Fig 1, for details see [34]). The lower Famennian deposits here investigated are 21 m thick and best outcrop in a trench (N50°47’43,476”, E20°33’53,568”). The deposits comprise thin-bedded, laminated, dark, carbonaceous shales and thin-bedded, grey, micritic limestones, which stratigraphically encompass the *Palmatolepis crepida* standard conodont Zone [34]. The interval investigated is confined to lithologic unit H-4 of Racki & Szulczewski [35].

The specimen was investigated using an environmental scanning electron microscope (ESEM) under low vacuum conditions in uncoated state using back-scattered electron (BSE) imaging. The elemental composition was measured using an energy dispersive spectroscopy (EDS) detector coupled with the ESEM. The specimen is housed at the Faculty of Earth Sciences, Sosnowiec, abbreviated GIUS 4–3657.

Dyckerhoff Polska Sp. z o.o. granted permission for fieldwork in the Kowala quarry.

## Results

**Fig 2:**
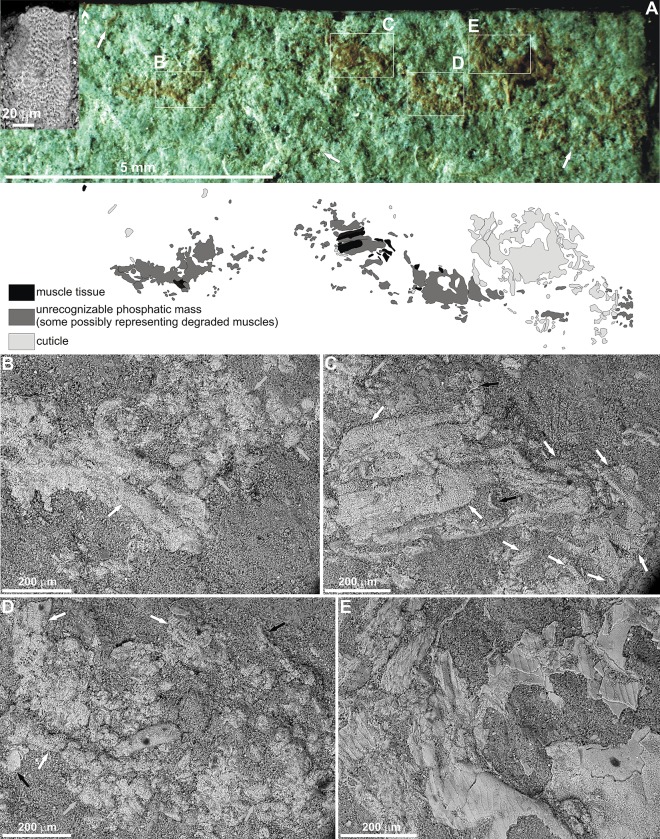
A. Surface of an Upper Devonian marly shale from the Kowala quarry with scattered remains. Arrows point to scattered remains outside the main patches. Line drawing indicating the position of particular remains is presented below. B-D. Variously preserved muscle tissues. White arrows indicate well-preserved tissues, grey arrows indicate putative degraded muscles, and black arrows point to cuticle fragments. E. Cuticle fragments.

The structure containing preserved soft tissues is small, with a length of one cm exposed on the surface of a marly shale, and it is irregularly distributed. Its width varies from 0.5 mm to 1.3 mm (Fig 2A) and is formed by a few distinct patches of preserved material; however, some portions are loosely scattered around. The structure is light-brown in colour.

**Fig 3:**
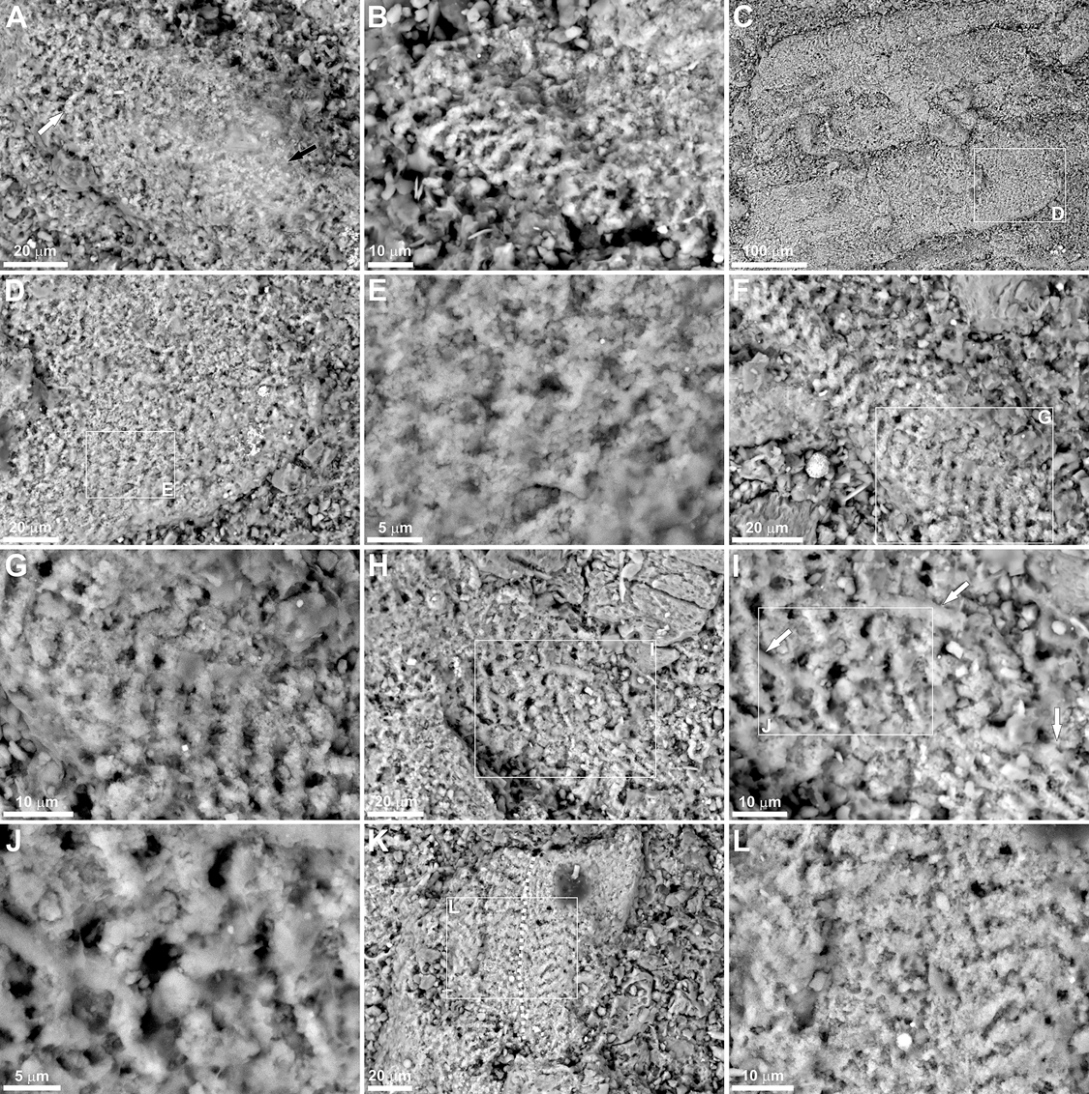
Magnifications of selected muscle tissues. A-B. Poorly preserved muscles with indistinct banding (white arrow) and degraded/recrystallized parts (black arrow). C-L. Well-preserved muscles showing well-developed banding. Arrows in I point to T-tubules-like structures. Tiny apatite crystallites replicating the muscle tissue are easily visible in magnified views E and J. White dotted line in K indicates the possible boundary between particular muscle fibers.

**Fig 4:**
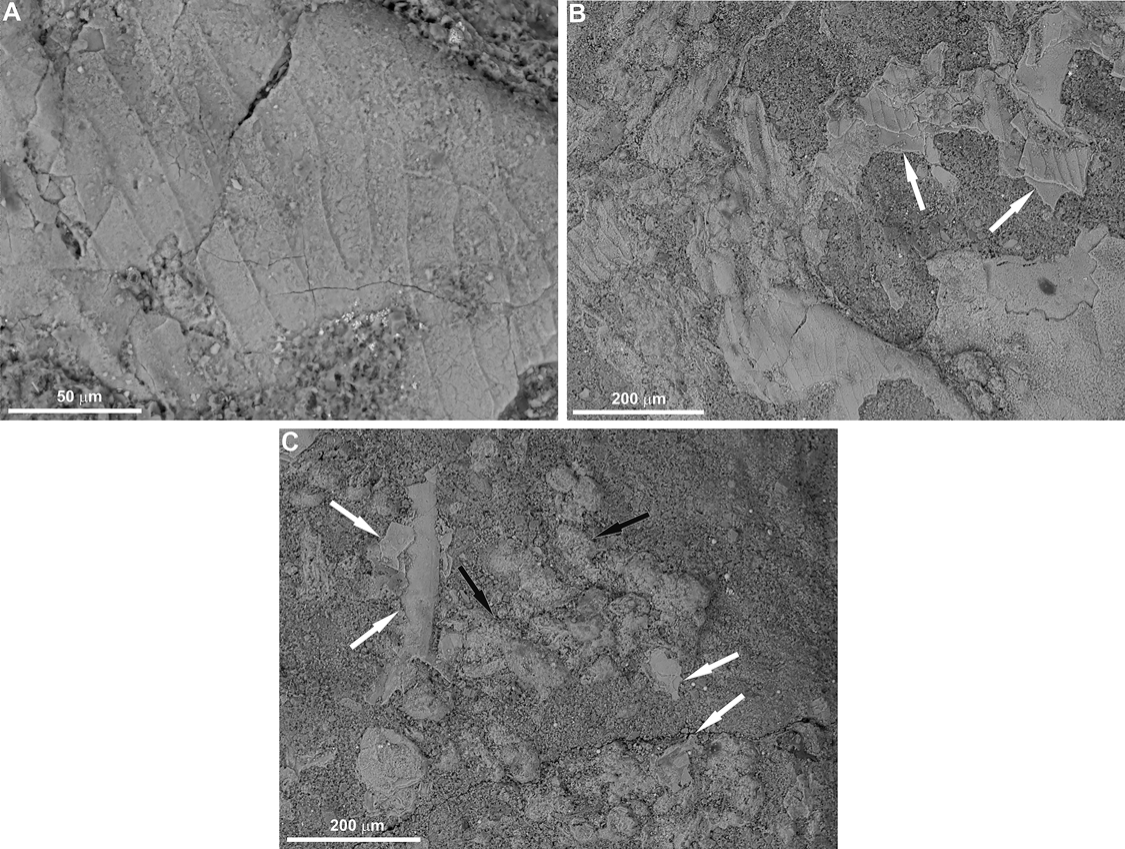
ESEM photomicrographs of cuticle fragments occurring close to the phosphatized muscle tissues from the lower Famennian of the Kowala quarry. A. View on a cuticle having distinct ornamentation in the form of ridges on its surface. B. Cuticle fragments showing layering by exfoliation of particular laminae (white arrows). C. Fragmented and dislocated cuticle fragments (white arrows) associated with putative highly degraded and/or recrystalized muscle tissue (black arrows).

The texture of the soft tissues reported here are comparable to muscle tissues preserved in fossil fishes and crustaceans [4, 16, 19, 27–28], as well as modern crustaceans subjected to decay experiments [29]. Like other fossil examples, some reported here are presumably also preserved in the form of bundles of fibers (Figs 2C and 3) which, in well-preserved specimens, show (typical for striated muscles) clear banding caused by separation of the sarcomeres (Fig 3C–3L). Although the muscle tissues retain their three-dimensional architecture, they are preserved differently. In one place they may be highly degraded and/or recrystallized as their structure is barely visible (Figs 2B, 3A and 3B), whereas in other places their state of preservation is very good (Figs 2A, 2C, 2D and 3C–3L). It is clear that the preserved muscle tissues and putative degraded muscles are fragmented and variously oriented on the bedding plane (Fig 2B–2D). In more poorly preserved specimens the banding is indistinct but still recognizable (Fig 3A and 3B). In some muscles, straight, tubular and sometimes branching structures c. 3μm in diameter occur (Fig 3I). Although incomplete, with respect to morphology and size, they could represent remnants of transverse tubules (T-tubules), organelles that have previously been reported in other phosphatized muscle tissues [28]. The phosphatized muscle tissues, both well-preserved and degraded, have been noted in patches (Fig 2B–2D); however, some isolated fragments have also been detected on the outside of the main fossilized mass (inset in Fig 2A). The remains preserved in the right, distal most part of the specimen primarily consist of differently preserved arthropod cuticle (Figs 2E and 4A). The arthropod affinity of the cuticle preserved is supported by laminated structure as evidenced from its exfoliated layers, as well as a characteristic external ornamentation (Figs 2E and 4B). In some places, the cuticle fragments are distinctly displaced and associated with structures which may represent highly degraded and/or recrystalized muscles (Fig 4C). Some cuticle fragments are also dispersed and associated with muscle tissues in other patches (Fig 2C–2D). The cuticle is, however, too fragmentary and lacks diagnostic features to be determined with confidence. In the lower Famennian of the Kowala quarry, three arthropod groups have been reported: thylacocephalans, phyllocarids and angustidontids [34]. Thylacocephalans may be excluded as those occurring in these deposits have a distinctive, polygonal ornamentation of the carapace. Angustidontids possess characteristic, granular ornamentation, at least on some parts of the carapace, and thus does not match the ornamentation present in the preserved cuticle fragments. Quite similar carapace ornamentation occurs in some phyllocarid crustaceans [36]; however, any complete specimen having such ornamentation has yet to be found in these deposits, but we can assume that the cuticle fragments belonged to a crustacean-like arthropod.

**Fig 5:**
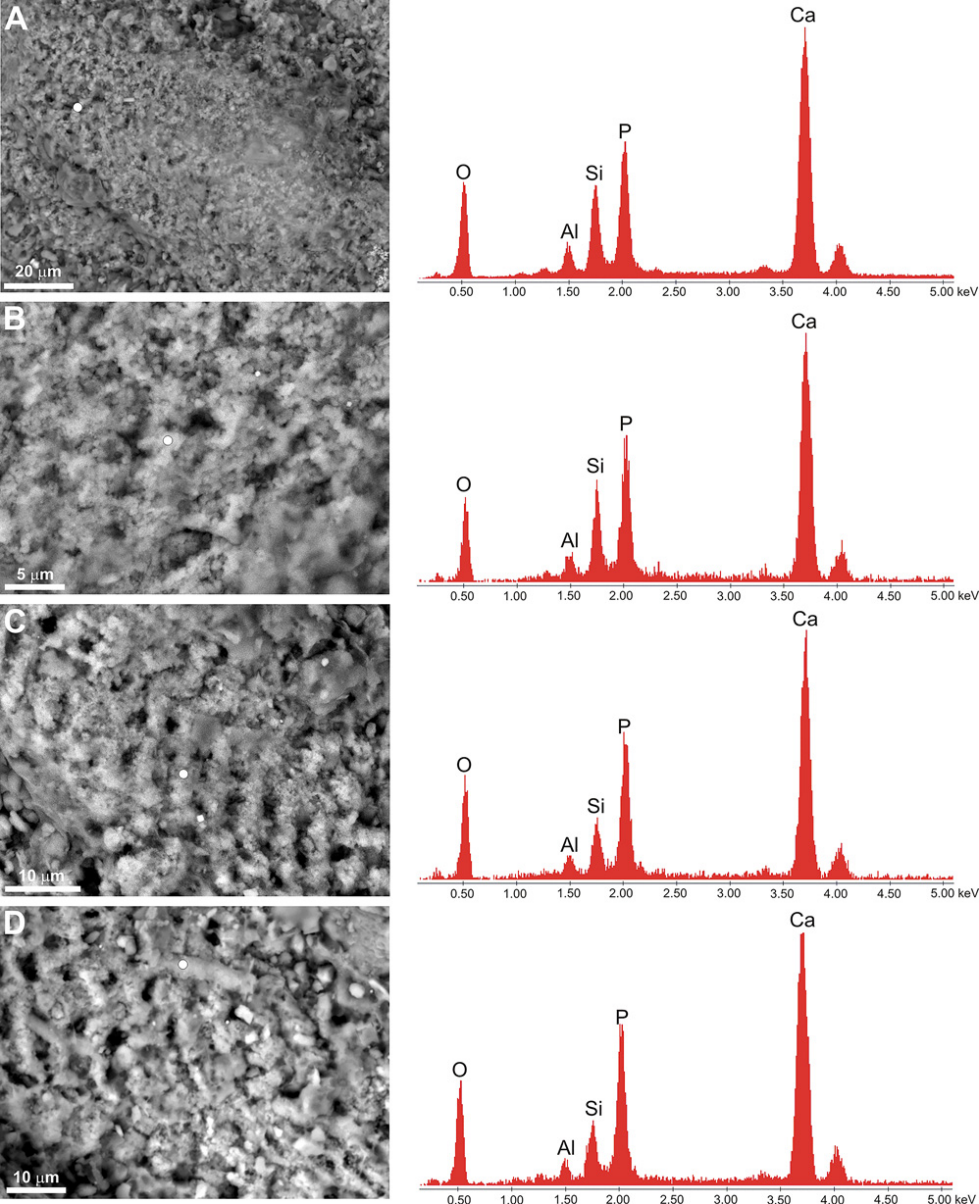
Spectra of EDS analysis of selected muscle tissues (A-C) and putative T-tubules structures (D), showing their phosphatic composition. The enrichment of aluminosilicates (Al, Si) is derived from the host sediment. White dots on the ESEM photomicrographs indicate the spots of the EDS analysis.

As shown by the EDS analysis, the preserved muscles tissues, as well as putative T-tubules, are phosphatic (see Fig 5). The phosphatized muscle tissues are characterized by the presence of tiny (0.7μm) apatite crystallites replacing the original tissue (Fig 3E and 3J). There is no direct evidence of the former presence of bacteria on the muscle tissues in the form of either phosphatized coccoid microstructures or hollows after bacterial cells preserved in phosphatized tissues [4, 16]. Thus, the phosphatized tissues here described are characterized by *substrate microfabric* sensu Wilby and Briggs [4].

## Discussion

The phosphatized muscles and cuticle most probably belong to the same animal, as there is no admixture of other fossils such as fish scales or teeth. Thus, the arthropod origin of the remains preserved is most likely. The context in which the described muscle tissues and associated cuticle are preserved indicates that they do not represent the remains of a buried carcass. The remains are fragmented, occur in patches and some are scattered around (Fig 2). Thus, they must represent only the small portion of the animal, incompletely preserved due to some biological and taphonomic processes. Although the interpretation of the fossilized remains is equivocal, the following three hypotheses concerning their origination are worth of discussion: 1. They represent the remains, not completely eaten by a scavenger or predator; 2. They represent the remains regurgitated by a predator, or 3. They represent the defecated remains preserved in the form of disaggregated coprolite.

**Fig 6:**
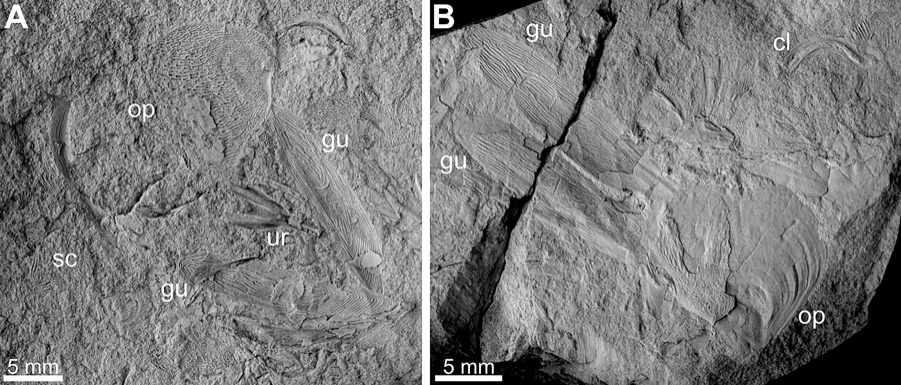
Coelacanth fish remains from lower Famennian deposits of the Kowala quarry. Remains of the head region of the specimens GIUS 4-3655/3 (A) and GIUS 4-3655/1 (B). Abbreviations of the elements identified: cl–cleithrum, gu–gulars, op–operculum, sc–scales, ur–urohyal.

The first hypothesis is supported by the preservation of the remains in the form of patchily distributed muscles and cuticle remains which are also chaotically scattered around. Additionally, they are fragmentary which is suggestive for scavenging on an arthropod by other arthropods or even fish. Although other, unpreserved animals could have been responsible, both of the latter organisms have good fossil record in these deposits. Potential scavenging arthropods may have been represented by thylacocephalans, phyllocarids or angustidontids [34]. The fishes are well-represented by isolated shark teeth [37] and recently discovered coelacanth remains (Fig 6). Moreover, indirect evidence for fish activity in the area of the present-day Kowala area during the early Famennian consists of numerous coprolites preserving both arthropod cuticle and small fish remains [38]. In the case of this hypothesis, the remains would represent the smaller, uneaten portions of the animal which were quickly buried within anoxic sediment. The laminated fabric of the host sediment [34] indicates that disintegration of the dead arthropod by the burrowing animals had not taken place.

The second hypothesis that the remains represent the regurgitated mass, expelled by the predator (most probably a fish) is also considered possible. The main problem, however, is the lack of any modern and fossil example of a regurgitate preserving soft tissue of the prey. Also the fragmented carapaces of thylacocephalan arthropods noticed from the same lower Famennian deposits and interpreted as fossilized regurgitates [39] are devoid of any signs of muscle tissue preservation. Known from both the Recent and the fossil record, the regurgitates mainly contain undigested hard parts in the form of bones of vertebrates or shells and other skeletal remains of invertebrates [40–44]. However, the occurrence of the remains in the form of patches and scattered elements around is quite suggestive for the regurgitate hypothesis. If true, to be preserved, the muscle tissues couldn’t have passed through the digestive tract of the fish, but had to be very quickly expelled out of the mouth after swallowing the prey. It is known that whereas the fossilized prey remains in the fish’s stomach may be very well-preserved, they do not show any traces of soft tissue preservation [45]. Thus, the very short residence time in the fish’s digestive tract would be a prerequisite for the preservation of the muscle tissue reported here. Such a rapid regurgitation of ‘fresh’ remains would even indicate that it was a non-habitual process resulting from, for example, irritation of the digestive tract. Besides a very short residence time of the soft tissues within the predator’s interior, such labile soft tissues as muscles would have been preserved only during special taphonomic conditions. These may have been established on the sea-floor where the remains were embedded within mucus. As a cohesive and microbe-bearing medium, the mucus could have separated the remains from the external environment and provided a suitable microenvironment for phosphatization. However, to be certain as they represent fossilized regurgitates preserving soft tissues, some comparative experiments involving modern fish and crustaceans would be desirable.

The third hypothesis is that the remains represent the disaggregated coprolite. In that case, the fragments preserved would represent the defecated remains of undigested tissues which passed through the predator’s digestive tract, following their disaggregation on the sea-bottom. Ironically, as in the fossil record there is no example of soft tissue preservation within the predator’s stomach [45] and fossilized regurgitates, there is one example of preservation of undigested muscle tissue preserved within the Late Cretaceous dinosaur coprolite [46]. The latter example is a unique, as it clearly shows that not all labile tissue were completely digested and some not only survived degradation within the animal’s stomach but also later on during the coprolite formation. This opens a possibility that such structures as coprolites may contain more paleobiological informations than previously thought. Fish coprolites are well-known from the lower Famennian deposits from the Kowala quarry either in the form of compact, pellet-like structures [38] and more disaggregated masses. However, despite the fact that they contain the prey’s hard parts (teeth, scales, cuticle), no any remnants of phosphatized soft tissue has been detected. Moreover, all the undigested remains in both the compact and disaggregated coprolites are enclosed within the phosphatic groundmass, which is absent in the present example. Thus, the aggregation of the soft tissues and cuticle fragments clearly differ with respect to taphonomic features from the coeval coprolites.

From the taphonomic features and comparisons presented above, it may be considered that the preserved muscles and cuticle remains do not represent the disaggregated coprolite, from which they substantially differ. The remains, however, may likely represent the leftover of scavenger activity on the arthropod animal, which subsequently were quickly buried. Such remains, but in the form of vertebrate bone fragments, are known from the fossil record [47]. The regurgitate hypothesis, although also likely, suffers from the lack of any modern and fossil comparative material showing soft tissue preservation.

The preservation of soft tissues in fossils are mainly limited to the Konservat-Lagerstätte deposits which formed under special environmental and geochemical conditions, where carcasses were not only unaffected by scavengers and bioturbators, but also were quickly buried and their labile soft tissues rapidly mineralized [2, 30, 48–49]. The lower Famennian deposits from the Kowala quarry which are rich in phosphatic cuticle of arthropods (thylacocephalans, phyllocarids and angustidontids) and carbonaceous compressions of non-biomineralized algae [34], have not provided any signs of animal soft tissue preservation until now. Thus, the present find indicates that in these deposits, at least locally, even soft tissues manipulated by a scavenger may be preserved when suitable conditions occur prior to and during burial. This increases the chances of finding more fossilized soft tissues in the deposits investigated here, as well as in other deposits originally known from the absence of soft tissue preservation.

In comparison to the Frasnian-Famennian boundary interval at the Kowala quarry, the deposition of the lower Famennian deposits of the *crepida* Zone took place in a more oxygenated sea-bottom conditions, as is evidenced from Th/U ratios [50], biomarkers and framboid pyrite size distributions [51]. Such conditions must have prevailed during scavenging on the arthropod remains on the sea-floor. Episodically, however, the environment witnessed dysoxia and anoxia on the sea-floor and even euxinia in the water column above, as documented by such specific biomarkers as aryl isoprenoids, isorenieratane and gammacerane [51]. The episodic, oxygen-deficient bottom waters are also indicated by large concentrations of amorphous organic matter [52], the impoverished benthic fauna, the common disseminated pyrite crystals and the presence of fossilized non-calcified algae [34]. Fluctuating redox conditions and the onset of oxygen-deficiency on the sea-bottom would have been the first step in preservation of the soft tissue remains by exclusion of further scavengers. After burial, the remains entered the closed, oxygen-deficient microenvironment devoid of bioturbators which otherwise would either directly degrade the tissues or indirectly influenced their degradation via oxygenated waters penetrating the sediment [2]. Indeed, the lack of bioturbators is well evidenced not only in the laminated fabric of soft tissue-bearing marly shale sample, but also in laminated marls and limestones occurring in some intervals of the studied deposits [50]. Such closed conditions within the sediment were crucial for preservation of the muscles, as it is well known that phosphatization of soft tissues requires special microenvironment characterized by specific pH and redox conditions, and the presence of sufficient concentration of phosphate [11, 20, 29–30]. The absence of fossilized microbes on the phosphatized muscle tissues reported here indicates that such *substrate microfabric* [4] may have resulted from autolytic decay that has created a favourable chemical environment characterized by the presence of sufficient phosphate availability. In such conditions, the muscle tissues may be preserved with high fidelity by extremely small apatite crystallites [4]. Although the phosphorous may have been delivered from both the decaying tissues and the sediment [4], in the present case it would have been derived from decaying carcass remains. Although the preserved remains consist of small bits of phosphatized soft tissues, it is supported by the laboratory experiments of Briggs and Kear [29], which clearly demonstrated that phosphatization of soft tissues do not need very high concentrations of phosphate external to the carcass. Moreover, the weakly (Fig 3A and 3B) and poorly preserved muscles without any signs of banding (Fig 2B) may suggest that these, during decay, delivered much of phosphate used for mineralization of those muscles which are here best preserved (Figs 2A, 2C, 2D and 3C–3L). This is also supported by the laboratory experiments [29] which showed that phosphate liberated from the decaying soft tissues of a shrimp, was used for mineralization of the adjacent muscle tissue.

## Conclusions

The phosphatized muscle tissues associated with cuticle fragments of arthropod origin have been detected in the lower Famennian of the Holy Cross Mountains, Poland, for the first time. The taphonomic features of the preserved remains indicate, that they did not belong to a complete animal buried within the sediment following its death. The taphonomic features and comparisons show that the remains do not represent fossilized fecal material (coprolite). They could have resulted from regurgitation of undigested remains by a predatory fish; however, the lack of any comparative data from both modern settings and the fossil record, makes the regurgitation hypothesis equivocal. Rather, the fragmentary nature of the specimen indicates, that the preserved remains represent a leftover after arthropod or fish scavenging on an arthropod, which were subsequently quickly buried in a suitable microenvironment allowing for their rapid phosphatization. This single example of phosphatized muscle tissues indicates that soft tissues, even earlier manipulated by scavenger, may be preserved if only special microenvironmental and taphonomic conditions within and around the animal remains are established. The present find makes a chance to find more fossilized soft tissues in the deposits investigated here, as well as in other deposits originally known from the absence of soft tissue preservation.
